# miR-25 inhibits sepsis-induced cardiomyocyte apoptosis by targetting PTEN

**DOI:** 10.1042/BSR20171511

**Published:** 2018-04-13

**Authors:** Yulong Yao, Fangyuan Sun, Ming Lei

**Affiliations:** Department of Critical Care Medicine, The Seventh People’s Hospital of Shanghai University of Traditional Chinese Medicine, Shanghai 200120, People’s Republic of China

**Keywords:** cardiomyocyte apoptosis, miR-25, PTEN, Sepsis, TLR4/NF-κB

## Abstract

To investigate the regulatory mechanism of miR-25 in sepsis-induced cardiomyocyte apoptosis. Rats models of sepsis were established by cecal ligation and puncture (CLP). Lipopolysaccharide (LPS)-induced cardiomyocyte was used as an *in vitro* model of sepsis. The expressions of miR-25, tensin homolog deleted on chromosome 10 (PTEN), Toll-like receptors 4 (TLR4), and p-p65 were analyzed by quantitative real-time PCR (qRT-PCR) and Western blot, respectively. The levels of interleukin-6 (IL-6) and tumor necrosis factor-α (TNF-α) were detected by ELISA. Cell apoptosis was detected by terminal deoxynucleotidyl transferase-mediated d-UTP nick end labeling (TUNEL) assay. The relationship between miR-25 and PTEN was measured by luciferase reporter assays. MiR-25 expression in serum of CLP rats and LPS-induced cardiomyocyte was decreased, while the contents of TNF-α and IL-6 were increased. Moreover, the expressions of PTEN, TLR4, and p-p65 in LPS-induced cardiomyocyte were significantly increased. Overexpression of miR-25 increased the survival rate of rats, inhibited LPS-increased cardiomyocyte apoptosis, reversed the increased expression of PTEN, TLR4, p-p65, TNF-α, and IL-6 induced by LPS. The luciferase assay demonstrated that PTEN was a target of miR-25. Additionally, pcDNA-PTEN reversed the inhibitory effect of miR-25 mimic on cardiomyocyte apoptosis, while TAK-242 (TLR-4 inhibitor) countered this effect. miR-25 reduced LPS-induced cardiomyocyte apoptosis by down-regulating PTEN/TLR4/NF-κB axis.

## Introduction

Sepsis, an infection-induced systemic inflammatory disorder, can lead to multiple organ dysfunction syndrome [1]. Although some results have been achieved in the prevention and treatment of sepsis in recent years, sepsis remains the main cause of mortality in the intensive care unit (ICU) [2,3]. Sepsis-induced myocardial dysfunction (SIMD) is one of the reasons leading to increased mortality in sepsis, but the pathogenesis remains unclear. Therefore, it is important to explore its pathogenesis in the diagnosis, prevention, and treatment of sepsis.

miRNAs are a class of endogenous non-coding RNA with the length of 21–25 nts, which can participate in the regulation of post-transcriptional gene expression, cell proliferation, differentiation, and inflammatory response [4]. Now, many researchers have found that miRNAs are associated with SIMD. Wang et al. [5] found that *miR-21-3p* could promote myocardial injury induced by sepsis. Zhou et al. [6] reported that *miR-155* alleviated myocardial injury in septic rats by inhibiting JNK-related inflammatory signaling. *MiR-25* has been found as an inhibitor for cardiomyocyte apoptosis induced by hypoxia/re-oxygenation [7], which suggest that *miR-25* may be related to myocardial function. Previous studies have also found that *miR-25* expression is down-regulated in patients with sepsis [8,9]. However, whether *miR-25* is involved in the pathogenesis of SIMD remains unknown.

Tensin homolog deleted on chromosome 10 (PTEN) is a double-specific phosphatase, which can be used as a natural antagonist of phosphatidylinositol 3-kinase (PI3K) [10]. Studies found that PTEN could participate in the regulation of lipopolysaccharide (LPS)-induced Toll-like receptors 4 (TLR4) signaling pathway and inflammatory response [11]. It has been proved that TLR4-mediated innate immunity and inflammatory response play an important role in the dysfunction of cardiomyocyte caused by sepsis [12]. Therefore, it may be speculated that PTEN may be associated with SIMD.

In the present study, we determined the expression of miR-25 and PTEN in different treated groups and detected how miR-25 regulated PTEN, and their effects on cardiomyocyte apoptosis. It might provide a theoretical basis for the development of new sepsis treatment.

## Methods

### Animals

Fifty-six SD rats (male, 220 ± 20 g) were purchased from Shanghai University of Traditional Chinese Medicine. Fourteen of them were randomly divided into two groups (*n*=7), namely sham group and cecal ligation and puncture (CLP) group. The others were separately divided into pre-NC group and miR-25 mimic group (*n*=21), and both groups of rats were divided into three subgroups (*n*=7) at different time points after the operation (24, 48, and 72 h). Rats in miR-25 mimic group were treated with miR-25 mimic (10 μl) by tail intravenous injection.

### Sepsis model

Sepsis model was established by CLP, as detailed previously (Hubbard et al. [13]). Briefly, rats were fasted for 12 h and water deprived for 4 h before operation. Then, rats were anesthetized by intraperitoneal injection of 10% chloral hydrate solution (0.35 ml/100 g). Anesthetized rats were received to a midline laparotomy, and the cecum was carefully isolated. The cecum was ligated using 4-0 silk suture and punctured twice using an 18-gauge needle. Meanwhile, a small amount of feces were extruded from the puncture site to ensure successful perforation. Then, the cecum was reset, and the abdominal cavity was closed. Rats in sham group did not undergo ligation and perforation. After operation, all the rats were subcutaneously injected with normal saline (5 ml/100 g). The post-operative shivering and activity decreased were observed in the control group, but all recovered within 24 h. Rats in CLP group showed severe sepsis 24 h after operation, such as hypothermia, drowsiness, pilo-erection, chills, activity, and diet reduction, which suggested that sepsis model was successful. Four weeks after the operation, rats were killed. The indexes of left ventricular function, including left ventricular systolic pressure (LVSP) and left ventricular end-diastolic pressure (LVEDP) were measured using rodent ultrasonography (PanoView b1500, Cold Spring Biotech Corp.).

### Cell culture and transfection

Cardiomyocytes (H9C2 cell line) were purchased from Cell Bank of Chinese Academy of Sciences. Cells were cultured in DMEM supplemented with 1.5 g/l NaHCO_3_, 10% FBS (Gibco, Invitrogen, U.S.A.), 1% glutamine, and 1% penicillin-streptomycin in 5% CO_2_ incubator under 37°C. H9C2 cells were treated with LPS for 24 h. MiR-25 mimic, miR-25 inhibitor, and pcDNA-PTEN were synthesized by GenePharma (Shanghai, China). Lipofectamine 2000 (Invitrogen) was used to perform cell transfection according to the manufacturer’s instructions.

### Quantitative real-time PCR

Total RNA from serum and H9C2 cells was extracted using TRIzol reagent (Invitrogen). cDNAs were synthesized using TaqMan miRNA Reverse Transcription Kit (Applied Biosystems, U.S.A.).

Quantitative real-time PCR (qRT-PCR) reactions were performed using ABI Prism 5700 Sequence Detection System (Applied Biosystems). U6 snRNA served as an internal normalized reference for miR-25, and β-actin used as an internal control for PTEN and TLR4. The 2^−ΔΔ*C*^_t_ method was used to calculated target genes expression.

### Western blot

Total protein was extracted from H9C2 cells using RIPA lysis buffer (Beyotime, Beijing, China). Proteins samples were then separated on SDS/PAGE (12%) and transferred on to PVDF membranes (Millipore, U.S.A.). The membranes were blocked with 5% skim milk for 60 min. Anti-β-actin, anti-PTEN, anti-TLR4, and anti-p-p65 were used as the first primary antibody at 1:1000 dilutions. The corresponding horseradish peroxidase-conjugated secondary antibody (Invitrogen) was incubated at room temperature for 1 h. Band intensity was quantitated by Quantity one software. The protein expression was normalized to β-actin levels.

### ELISA

Blood samples (5 ml) were taken from abdominal aorta and centrifuged at 3000 rpm for 15 min in 4°C. Cells from different groups were collected and centrifuged at 1500 rpm for 5 min. Then, the supernatant was collected. ELISA kit was used to measure the levels of tumor necrosis factor-α (TNF-α) and interleukin-6 (IL-6) following the manufacturer’s instructions.

### Terminal deoxynucleotidyl transferase-mediated d-UTP nick end labeling assay

Terminal deoxynucleotidyl transferase-mediated d-UTP nick end labeling (TUNEL) kit (Beijing Zhongshan Biotechnology Co.) was used to detect cell apoptosis. Briefly, H9C2 cells were fixed with 4% formaldehyde for 10 min after washing with PBS for three times. Then, cells were incubated with TUNEL solution at 37°C for 1 h. After that, cells were stained with DAPI for the observation of apoptosis.

### Luciferase reporter assays

H9C2 cells were seeded into 24-well plates at a density of 1 × 10^5^ cells/well and tested with luciferase reporter assay (Promega, U.S.A.) according to the manufacturer’s instructions. Briefly, the fragments of PTEN-3′-UTR containing *miR-204* putative binding region (3′UTR-WT) or mutant PTEN-3′-UTR containing miR-204 putative binding region (3′UTR-Mut) were inserted into a luciferase reporter gene plasmid (Invitrogen). Then, miR-25 mimic or miR-25 inhibitor and reporter plasmids were co-transfected into cells using Lipofectamine 2000 (Invitrogen) for 48 h. The luciferase activities were determined using a luciferase reporter assay system (Promega) according to the manufacturer’s instructions.

### Statistical analysis

SPSS 20.0 was used for the data analysis. Student’s *t* test was performed to test the differences between groups. All data were repeated as independent experiments at least three times and presented as mean ± S.D. *P*<0.05 was considered statistically significant.

## Results

### The expression of miR-25 was decreased in serum of septic rats

**Figure 1:**
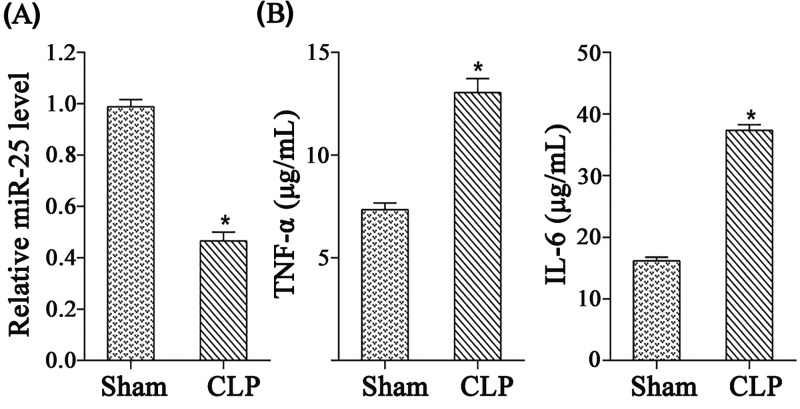
The expression of miR-25 was decreased in serum of septic rats Rats were divided into sham group and CLP group (*n*=7). Rats in CLP group were performed with CLP. (**A**) The relative expression of miR-25. (**B**) The expressions of TNF-α and IL-6. *P<0.05 compared with sham group.

Rats were divided into sham group and CLP group (*n*=7). Rats in CLP group were performed with CLP. As shown in Figure 1A, miR-25 expression in CLP group was reduced significantly compared with sham group. Contrarily, the expressions of TNF-α and IL-6 were markedly increased (Figure 1B). Moreover, the LVSP in CLP group was significantly decreased compared with the sham group (Supplementary Figure S1A), while the LVEDP was markedly higher in CLP group than in the sham group (Supplementary Figure S1B).

### MiR-25 regulated the survival rate and inflammation in rats

**Figure 2:**
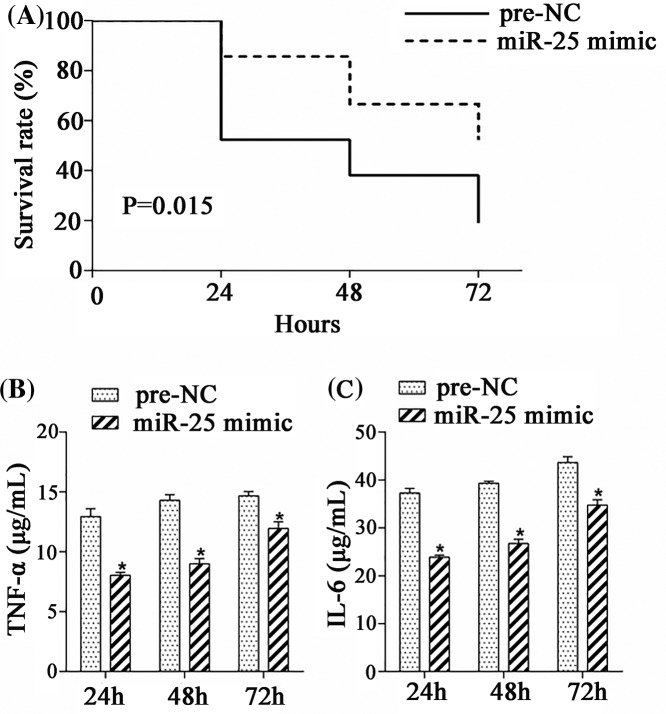
MiR-25 regulated the survival rate and inflammation in rats Septic rats were divided into pre-NC group and miR-25 mimic group (*n*=21). Rats in miR-25 mimic group were treated with miR-25 mimic (10 μl) by tail intravenous injection. After 24, 48, and 72 h, the survival rate was calculated, and the expressions of TNF-α and IL-6 in serum of rats were measured by ELISA. (**A**) The survival rate of rats. (**B**) The level of TNF-α. (**C**) The level of IL-6. *P<0.05 compared with pre-NC group.

Septic rats were divided into pre-NC group and miR-25 mimic group (*n*=21). Rats in miR-25 mimic group were treated with miR-25 mimic (10 μl) by tail intravenous injection. After 24, 48, and 72 h, the survival rate was calculated, and the expressions of TNF-α and IL-6 in serum of rats were measured by ELISA. Results showed that the survival rate in miR-25 mimic group was significantly higher than that in pre-NC group (Figure 2A). Meanwhile, the expressions of TNF-α and IL-6 in miR-25 mimic group were lower than that in pre-NC group at each time point (Figure 2B,C). These results indicated that miR-25 might regulate the sepsis process by inhibiting inflammation.

### MiR-25 expression was reduced in H9C2 cells induced by LPS

**Figure 3:**
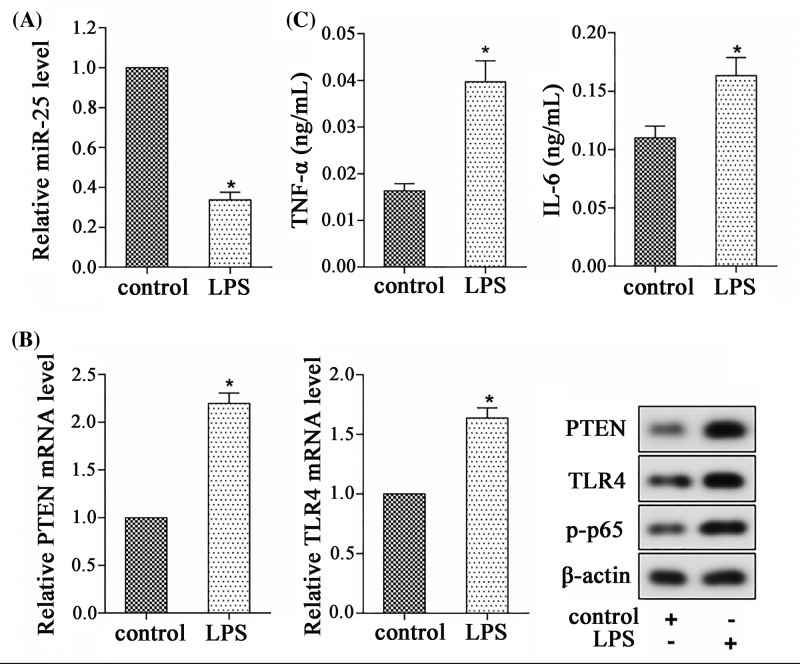
MiR-25 expression was reduced in H9C2 cells induced by LPS H9C2 cells in LPS group were treated with LPS for 24 h, and cells in control group were not given any treatment. (**A**) The relative expression of miR-25. (**B**) The mRNA and protein expressions of PTEN and TLR4. (**C**) The levels of TNF-α and IL-6. *P<0.05 compared with control group.

To investigate the effect of LPS on the expressions of miR-25, PTEN, and TLR4, H9C2 cells were treated with LPS for 24 h. The control group was not given any treatment. As shown in Figure 3A,B, LPS could down-regulate miR-25 expression and up-regulate the levels of PTEN, TLR4, and p-p65. Additionally, LPS increased the levels of TNF-α and IL-6 (Figure 3C). These results indicated that LPS might promote inflammation by repressing miR-25 expression, increasing PTEN expression and activating the TLR-4/NF-κB signaling pathway.

### MiR-25 regulated TLR-4/NF-κB pathway and inflammation

**Figure 4:**
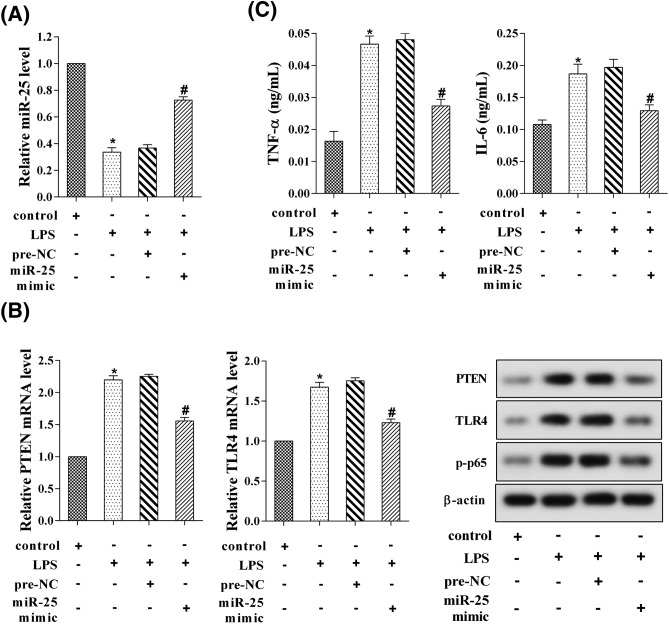
MiR-25 regulated TLR-4/NF-κB pathway and inflammation H9C2 cells were separately divided into four groups, namely control, LPS, pre-NC, and miR-25 mimic groups. (**A**) The relative expression of miR-25. (**B**) The mRNA and protein expressions of PTEN and TLR4. (**C**) The levels of TNF-α and IL-6. *P<0.05 compared with control; ^#^*P*<0.05 compared with LPS + pre-NC.

To investigate the role of miR-25 on TLR-4/NF-κB pathway and inflammation, H9C2 cells were separately divided into four groups, namely control, LPS, pre-NC, and miR-25 mimic groups. We found that LPS reduced the expression of miR-25, and overexpression of miR-25 reversed this effect (Figure 4A). Moreover, LPS increased the levels of PTEN, TLR4, and p-p65, and overexpression of miR-25 reversed this effect (Figure 4B). As shown in Figure 4C, LPS promoted the expressions of TNF-α and IL-6, and overexpression of miR-25 reversed this effect.

### MiR-25 directly targetted PTEN

**Figure 5:**
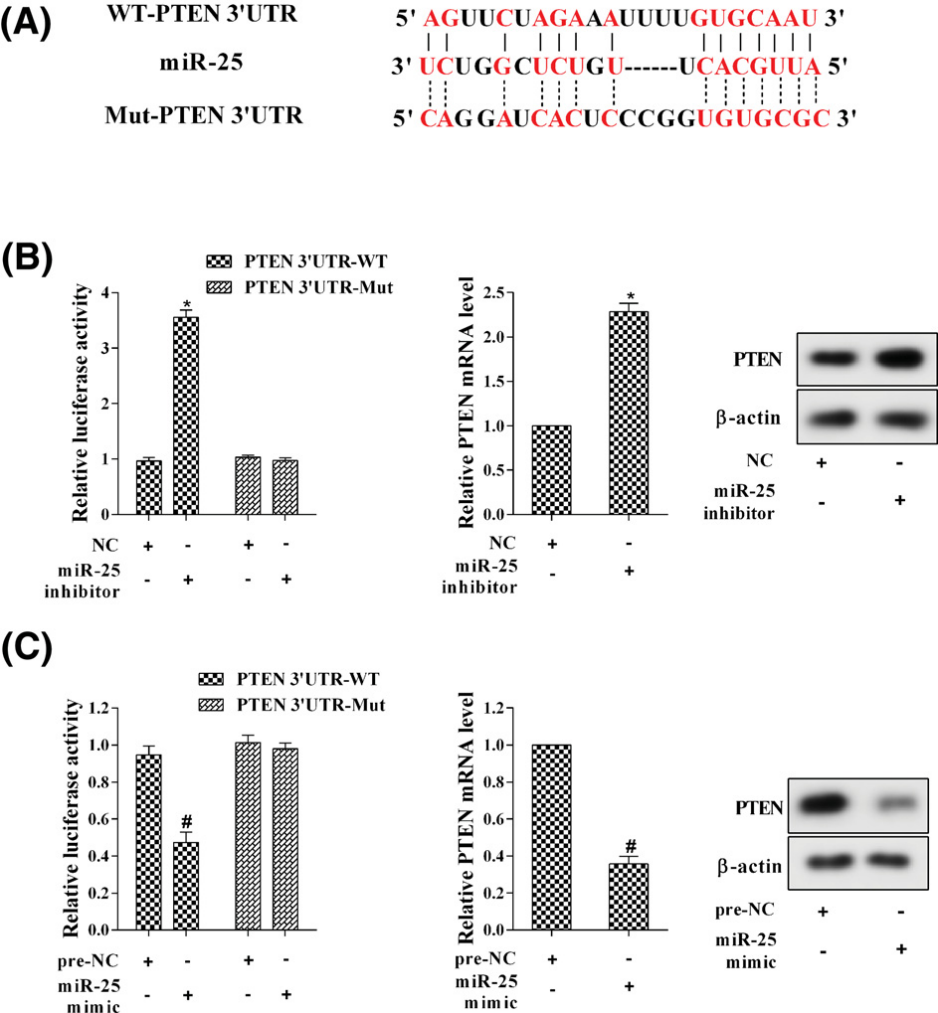
MiR-25 directly targetted PTEN (**A**) The bioinformatics analysis (microRNA.org). (**B**) The luciferase reporter activity and PTEN expression in cells transfected with miR-25 inhibitor. (**C**) The luciferase reporter activity and PTEN expression in cells transfected with miR-25 mimic. *P<0.05 compared with NC; ^#^*P*<0.05 compared with pre-NC.

To verify whether miR-25 directly targetted PTEN, luciferase reporter assay was performed. According to the bioinformatics analysis (microRNA.org), miR-25 could bind to 3′-UTR of PTEN (Figure 5A). Moreover, luciferase reporter assay demonstrated that miR-25 inhibitor significantly increased the luciferase reporter activity of PTEN 3′-UTR-WT and up-regulated the mRNA and protein levels of PTEN (Figure 5B). Contrarily, miR-25 mimic suppressed the luciferase reporter activity of PTEN 3′-UTR-WT and down-regulated the mRNA and protein levels of PTEN (Figure 5C). These findings proved that miR-25 could directly target PTEN.

### MiR-25 inhibited H9C2 cells’ apoptosis induced by LPS

**Figure 6:**
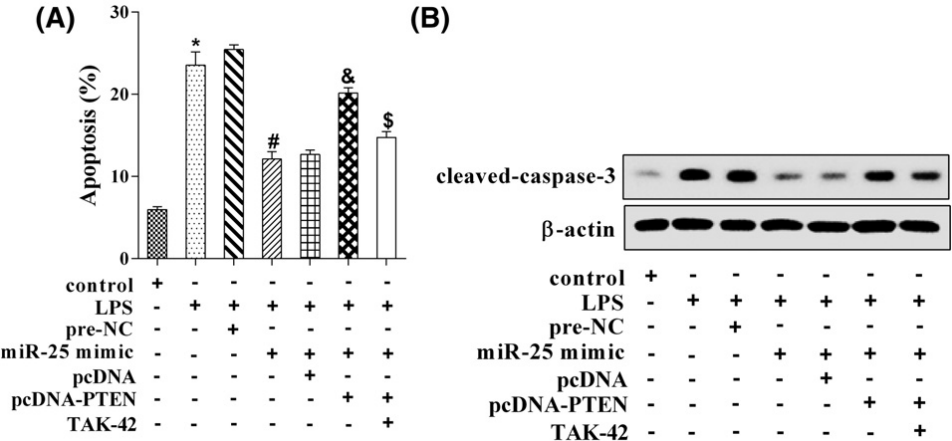
MiR-25 inhibited H9C2 cells’ apoptosis induced by LPS (**A**) LPS promoted H9C2 cardiomyocytes apoptosis, and miR-25 mimic could counter this effect. However, cell apoptosis increased markedly after adding pcDNA-PTEN, and TAK-242 (TLR-4 inhibitor, 1 μg/ml) treatment could reverse this effect. *P<0.05 compared with control; ^#^*P*<0.05 compared with LPS + pre-NC; ^&^*P*<0.05 compared with LPS + miR-25 mimic + pcDNA; ^$^*P*<0.05 compared with LPS + miR-25 mimic + pcDNA-PTEN. (**B**) The protein level of cleaved-caspase-3.

As shown in Figure 6A, LPS promoted H9C2 cardiomyocytes’ apoptosis, and miR-25 mimic could counter this effect. However, the cell apoptosis increased markedly after adding pcDNA-PTEN, and TAK-242 (TLR-4 inhibitor, 1 μg/ml) treatment could reverse this effect. Moreover, LPS up-regulated the protein level of cleaved-caspase-3, and miR-25 mimic down-regulated its protein level. After adding pcDNA-PTEN, the protein level of cleaved-caspase-3 was increased. As expected, TAK-242 countered the increased effect (Figure 6B).

## Discussion

SIMD is one of the major causes of deaths in patients with septic [14]. It was reported that the mortality rate of septic patients with myocardial dysfunction was 70%, and the mortality rate of septic patients without myocardial dysfunction was only 20% [15]. Therefore, early detection of SIMD and timely inhibition of the occurrence and development of the disease play an important role in promoting the rehabilitation and prognosis of patients with sepsis.

MiRNAs are involved in the regulation of physiological and pathological processes in multiple organ systems [16], including cardiovascular system [17]. Pan et al. [18] reported that miR-25 could protect cardiomyocytes from oxidative damage. Liu et al. [7] found that miR-25 could inhibit cardiomyocyte apoptosis. These suggested that miR-25 might be an important regulator in myocardial function. However, the mechanisms of miR-25 in sepsis have not been thoroughly revealed. Our study provided new evidences that miR-25 inhibited LPS-induced cardiomyocyte apoptosis by regulating PTEN and its downstream signaling pathway. LPS, an endotoxin, can cause excessive activation of the immune system, resulting in septic death [19,20]. Therefore, our study enriches the literature that supports miR-25 playing an important role in sepsis.

Additionally, we detected that miR-25 expression in serum of septic rats was significantly decreased, which was consistent with previous reports [8,9]. Moreover, miR-25 remarkably increased the survival rate of septic rats and inhibited inflammation. Thus, we speculated that miR-25 might be a meaningful biomarker for sepsis. To investigate the underlying mechanism of miR-25 in regulating the progress of sepsis, LPS-induced H9C2 cardiomyocytes were used as an *in vitro* model to study the apoptosis of cardiomyocytes induced by sepsis. We found that LPS could down-regulate miR-25 expression, up-regulate PTEN and activate its downstream signaling pathway (TLR-4/NF-κB pathway), while overexpression of miR-25 reversed the effects, which illustrated that miR-25 could inhibit PTEN expression and TLR-4/NF-κB pathway induced by LPS.

TLR-4/NF-κB pathway is one of the most important signaling pathways in the inflammatory response of sepsis [21]. Studies have shown that excessive activation of TLR4/NF-κB pathway may induce cardiomyocytes dysfunction [22,23]. TAK-242 is an inhibitor of TLR-4, which can alleviate inflammation and reduce cardiomyocytes apoptosis by inhibiting TLR4/NF-κB signaling pathway [24,25]. The current study confirmed this point. We found that TAK-242 could reverse the effect of pcDNA-PTEN on cardiomyocytes apoptosis. PTEN is a tumor suppressor gene with phosphatase activity, which plays a role in many physiological activities such as cell differentiation, apoptosis, and migration [26]. In this study, PTEN was confirmed as the target gene of miR-25 for the first time. Moreover, we found that overexpression of miR-25 could reverse the promoting effect of LPS on cardiomyocyte apoptosis, while overexpression of PTEN countered the inhibitory effect of miR-25 mimic on cardiomyocyte apoptosis. These results indicated that miR-25 could inhibit LPS-induced cardiomyocyte apoptosis by regulating PTEN.

Additionally, studies have shown that a large number of pro-inflammatory cytokines released in the early stage of sepsis can cause myocardial tissue injury [27]. The current study demonstrated that the levels of TNF- and IL-6 in the serum of septic rats and in LPS-induced cardiomyocytes were increased, while the levels were decreased significantly after transfecting miR-25 mimic, which indicated that miR-25 could alleviate myocardial injury caused by sepsis through inhibiting inflammatory reaction.

In conclusion, miR-25 might inhibit the activation of inflammatory cells by regulating PTEN/TLR4/NF-κB axis, thereby reducing the apoptosis of cardiomyocytes and relieving LPS-induced myocardial injury.

## Supporting information

**Supplement Figure 1:** The index of left ventricular function in rats. (A&B) LVSP and LVEDP were detected by cardiac ultrasound. *P<0.05 *vs* sham.

## Abbreviations

CLP: cecal ligation and puncture
DMEM: Dulbecco’s Modified Eagle Medium
IL-6: interleukin-6
JNK: c-Jun N-terminal kinase
LPS: lipopolysaccharide
LVEDP: left ventricular end-diastolic pressure
NC: negative control
LVSP: left ventricular systolic pressure
NF-κB: Nuclear factor κB
PI3K: phosphatidylinositol 3-kinase
PTEN: tensin homolog deleted on chromosome 10
SD: standard deviation
SIMD: sepsis-induced myocardial dysfunction
TLR4: Toll-like receptor 4
TNF-α: tumor necrosis factor-α
TUNEL: terminal deoxynucleotidyl transferase-mediated d-UTP nick end labeling
