# Supplementary material for: miR-25 inhibits sepsis-induced cardiomyocyte apoptosis by targetting PTEN
Source: Biosci Rep. 2018 Apr 13;38(2):BSR20171511. doi: 10.1042/BSR20171511 (PMC5897747; doi:10.1042/BSR20171511)
Supplement: Supplementary file 1 [file bsr20171511_Supp1.pdf]

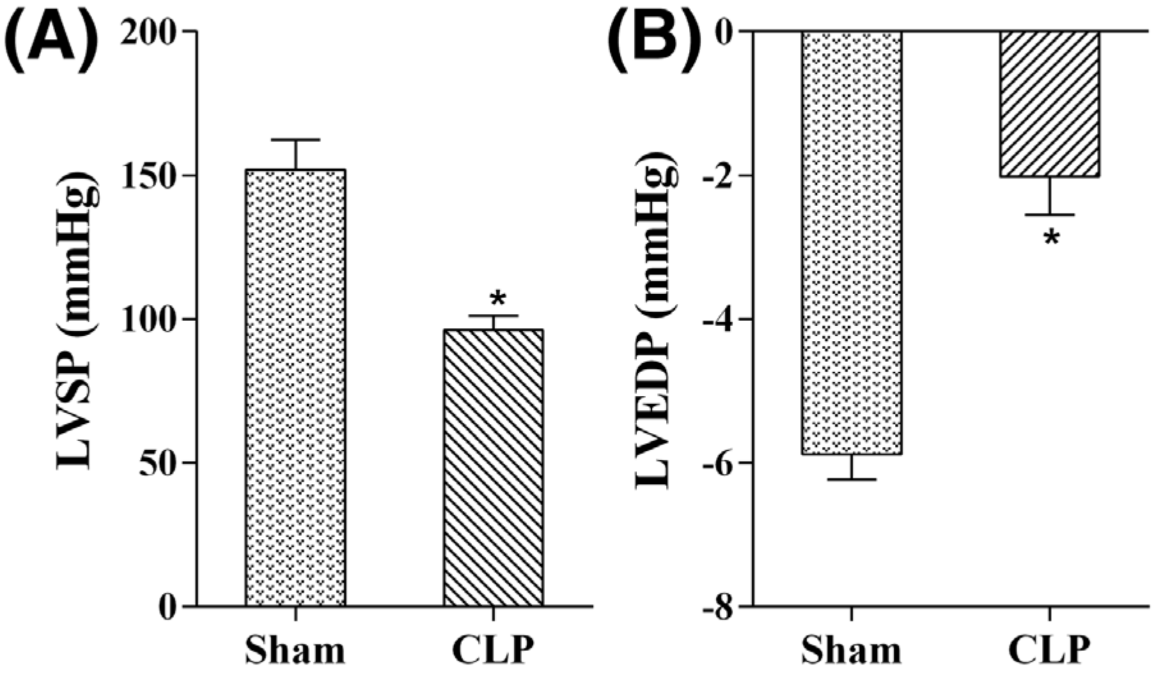

Supplement Figure 1: The index of left ventricular function in rats. (A&B) LVSP and LVEDP were detected by cardiac ultrasound. *P<0.05 *vs* sham.
